# Guiren Runchang granules alleviate slow transit constipation in mice by modulating gut microbiota and short-chain fatty acids

**DOI:** 10.3389/fmicb.2025.1615297

**Published:** 2025-09-29

**Authors:** Xinfeng Bai, Yachao Wang, Kai Wang, Weihua Chu, Tianyu Bai

**Affiliations:** ^1^Microecological Medicine Research Center, Shandong Provincial Third Hospital, Shandong University, Jinan, China; ^2^Department of Microbiology and Synthetic Biology, School of Life Science and Technology, China Pharmaceutical University, Nanjing, China

**Keywords:** traditional Chinese medicine, Guiren Runchang granule, constipation, gut microbiota, SCFAs

## Abstract

Guiren Runchang Granules (GRG), a traditional Chinese medicine (TCM) formulation, has demonstrated effectiveness in treating slow transit constipation (STC). This study investigated the therapeutic effects of GRG on constipated mice, focusing on its role in restoring intestinal micro-ecology. The results showed that GRG treatment significantly improved defecation in STC mice and ameliorated intestinal histopathological damage. GRG effectively normalized colonic and serum motility-related hormone levels (5-HT, SP, MTL, and SS) with comparable efficacy to mosapride, a prokinetic agent. In addition, GRG treatment restored the balance of the gut microbiota and increased the levels of short-chain fatty acids (SCFAs). Specifically, the relative abundance of the genera *Alloprevotella* and *Muribaculum* increased, while that of *Prevotellaceae_NK3B31_group*, *Ruminococcus*, and *Lachnospiraceae_UCG-001* decreased. These microbiota changes were significantly correlated with SCFAs level, motility-related hormone concentrations, and defecation phenotype. Our findings highlight GRG’s multi-target mode of action involving microbiota modulation, SCFAs restoration, and hormonal regulation, positioning it as a promising alternative to conventional therapies for STC.

## 1 Introduction

Functional constipation, a prevalent gastrointestinal disorder affecting over 15% of the global population (Barberio et al., 2021), imposes significant socioeconomic burdens and compromises quality of life (Harris and Chang, 2022). Chronic constipation can damage the intestinal wall and promote the accumulation of harmful substances and pathogenic bacteria, which are critical risk factors for colorectal cancer (Song et al., 2021). Constipation is also associated with an increased risk of cardiovascular disease, Parkinson’s disease, and depression (Sumida et al., 2019).

The pathogenesis of STC involves intricate interactions among neurological disorders, hormonal imbalances, smooth muscle dysfunction, abnormal interstitial cells of Cajal (ICCs), and disruptions in the gut microbiota (Bharucha and Lacy, 2020). The gut microbiota plays a central role in regulating gastrointestinal physiology and maintaining intestinal function (Fan and Pedersen, 2021). The gut microbiota contributes to suppressing pathogen overgrowth, maintaining the integrity of the mucosal barrier (Martens et al., 2018), and supporting the development of the enteric nervous system (Vicentini et al., 2021). Moreover, bacterial metabolites such as SCFAs, bile acids, and tryptophan derivatives are closely linked to intestinal integrity and motility (Bhattarai et al., 2018). Accumulating evidence positions the gut microbiota dysbiosis as a major risk factor for constipation (Ohkusa et al., 2019). Clinical evidence showing abnormal gut microbiota profiles in constipated patients (Shin et al., 2019), suggesting microbiota-directed therapies could offer novel therapeutic avenues.

Current pharmacological interventions, mainly including osmotic laxatives, secretagogues, and prokinetic agents (Pannemans et al., 2020; Gao et al., 2022), primarily target symptom relief but exhibit limited long-term efficacy and safety concerns, such as electrolyte imbalance and tachyphylaxis effects (Vriesman et al., 2020). TCM formulations, characterized by multi-component synergism, have been widely used to treat digestive disorders (Wang et al., 2022). Unlike conventional monotherapies, TCM exerts holistic effects by modulating gut microbiota, enhancing beneficial intestinal metabolites, promoting intestinal mucosal secretion, and protecting the epithelial barrier. GRG, a TCM compound, has shown clinical efficacy in alleviating STC symptoms. Previous studies attribute GRG’s activity to aquaporin modulation and ICCs protection (Sun et al., 2020). However, its interplay with the microbiota remains unexplored. This study established a loperamide-induced STC mouse model to evaluate the therapeutic effects of GRG, focusing on its effects on intestinal motility, gut microbiota, and SCFAs production.

## 2 Materials and methods

### 2.1 Chemicals and reagents

Guiren Runchang granules were prepared by the Traditional Chinese Medicine Pharmacy of Shandong Provincial Third Hospital, following previously published methods (Sun et al., 2020). GRG is a compound TCM formula composed of 10 medicinal materials, as detailed in Supplementary Table S1. Loperamide hydrochloride was obtained from Xi’an Yang Sen Pharmaceutical Co. Ltd. (Xi’an, China), and mosapride citrate was purchased from Shandong New Time Pharmaceutical Co., Ltd. (China). Kits for enzyme-linked immunosorbent assays (ELISA), specifically for motilin (MTL), substance-P (SP), somatostatin (SS), vasoactive intestinal peptide (VIP), and serotonin (5-HT), were sourced from Cusabio Biotech (Wuhan, China). SCFA standards (acetic acid, propionic acid, butyric acid, isobutyric acid, valeric acid, isovaleric acid, and hexanoic acid) were procured from Sigma-Aldrich Co. LLC (Shanghai, China). India ink was obtained from Sangon Biotech (Shanghai, China). All additional reagents and chemicals employed in this study were of analytical grade.

### 2.2 Animal experiment

Male BALB/c mice (6 weeks old, weighing 18–20 g, Jinan Pengyue Experimental Animal Co., Ltd., Jinan, China) were housed in a specific pathogen-free environment at 20–25°C with 40–60% relative humidity and a 12-h light/dark cycle. During the experiment, the mice were provided with unrestricted access to both food and water.

After a week of acclimation, 60 mice were randomized into six groups (10 mice per group): the normal control (NC), STC model, mosapride-treated positive control (MO), and low-dose (LD), medium-dose (MD), and high-dose (HD) GRG treatment groups. Mice within each group were housed in two cages (5 mice per cage). The STC model was established as previous study (Liu et al., 2023). All groups except NC were administered 10 mg/kg loperamide daily for 14 days. Therapeutic interventions commenced the day after model establishment and continued for 14 days. The dosages of GRG for intragastric administration were determined by converting the standard human clinical dose of GRG to a mouse equivalent dose (MED) using body surface area normalization. The LD, MD, and HD groups were treated by gavage with 4.72 g/kg/d, 9.44 g/kg/d, and 18.88 g/kg/d GRG, respectively, which were comparable to adult clinical sub effective, equivalent, and super therapeutic doses (Sun et al., 2020). The MO group received mosapride suspension, and the NC and STC groups were treated with saline.

On day 36, fresh fecal samples were firstly collected for microbiota and SCFA analysis. Mice were then transferred to clean cages to collect 1 h fresh feces and 12 h feces for defecation parameters. Subsequently, mice underwent a 12 h fasting period. Intestinal motility tests were performed on day 37, and mice were then euthanized under anesthesia for serum and colon tissue collection. Subsequently, these samples were subjected to experimental test.

### 2.3 Measurement of defecation parameters

After daily gavage, fresh feces produced over 1 h were collected, and their wet and dry weights were recorded. Fecal water content was calculated: Water content of the feces (%) = (wet weight-dry weight)/wet weight × 100% and the total feces produced during this period were collected for further analysis.

### 2.4 Determination of intestinal motility

Intestinal motility in mice was evaluated utilizing a small intestine transit rate test and a first black defecation experiment. Post 12 h fasting, 2 mice per cage (4 mice/group) were randomly selected using stratified sampling for the small intestine transit rate test. Each mouse received 0.2 mL of India ink via oral gavage. After 30 min, four mice were euthanized, and the small intestines were excised from the pylorus to the cecum. The intestinal transit rate was calculated: Intestinal transit rate (%) = transited distance by the Indian ink/total length of the small intestine × 100%.

Subsequently, another 2 mice per cage (4 mice/group) were randomly chosen for the first black defecation experiment. The mice were individually placed in clean cages to record the time to first black defecation.

### 2.5 Measurement of serum and colon hormones

Blood samples were obtained through retro-orbital collection following anesthesia. The samples were allowed to clot at ambient temperature for 2 h, then centrifuged at 3000 × g for 15 min at 4°C to separate the serum. The proximal colon tissue was dissected, washed with saline, and divided into three parts: two portions were stored at −80°C for hormone analysis and RNA extraction, while the third portion was fixed in 4% paraformaldehyde.

The contents of the MTL, SP, SS, VIP and 5-HT in the serum and colon samples Serum and colonic concentrations of MTL, SP, SS, VIP, and 5-HT were determined using a commercially ELISA kits.

### 2.6 Histopathological examination

Colon tissue sections were evaluated by independent pathologist blinded to the experimental group assignments. Fixed colon tissues were used to assess morphological changes. The tissues were dehydrated for 24 h, embedded in paraffin, sectioned, and stained with hematoxylin and eosin (H&E). Histological features were examined under an optical microscope.

### 2.7 16S rRNA sequencing and fecal microbiota analysis

The composition of fecal microbiota was analyzed by 16S rRNA gene-based amplicon sequencing method. Bacterial DNA was extracted from fecal samples using the ZR Fecal DNA Extraction Kit (Zymo Research, CA, USA). The variable V4 region of the 16S rRNA gene was amplified with barcoded specific bacterial primers 515F (5′-GTGCCAGCMGCCGCGGTAA-3′) and 806R (5′-GGACTACHVGGGTWTCTAAT-3′). The amplicons were purified utilizing a DNA gel extraction kit (OMEGA, USA). Sequencing libraries were prepared employing the TruSeq® DNA PCR-Free Sample Preparation Kit (Illumina, USA). Paired-end sequencing was performed on an Illumina HiSeq 2,500 platform (Illumina Inc., San Diego, CA, USA). Sequencing data processing and microbial composition analysis were conducted as previously described (Jia et al., 2024). QIIME II software (version 1.9.1) was used for data processing and analysis. The raw data underwent denoising to generate amplicon sequence variants using the DADA2 algorithm.

Community richness and diversity were quantified using the Chao1 and Shannon indices, respectively. To evaluate the relationships between samples, principal coordinate analysis (PCoA) was performed, providing insights into sample clustering in a low-dimensional space. Differential species were identified using t-test analysis. Microbial metabolic functions were inferred using PICRUSt 2, which maps species composition from 16S rRNA sequencing data to Kyoto Encyclopedia of Genes and Genomes (KEGG) metabolic pathways.

### 2.8 Quantification of SCFAs in fecal samples

The levels of SCFAs, including acetic acid, propionic acid, butyric acid, isobutyric acid, valeric acid, isovaleric acid, and caproic acid, in fecal samples were measured utilizing gas chromatography–mass spectrometry. Immediately after collection, fecal samples were promptly frozen and preserved at −80°C. Approximately 30 mg of each sample was suspended in 900 μL of 0.5% phosphoric acid and homogenized with a tissue grinder for 3 min. After centrifugation at 10,000 × *g* for 10 min, the clear supernatant was collected. An 800 μL aliquot of the supernatant was combined with an equal volume of ethyl acetate, vortexed for 5 min, and then centrifuged at 14,000 × *g* for 10 min. A 600 μL aliquot of the organic phase was extracted for analysis. SCFA concentrations were determined employing an Agilent 7890A/5975C GC–MS system (Agilent Technologies, USA) equipped with a DB-WAX capillary column (30 m × 0.25 mm, ID × 0.25 μm). Quantification was performed by comparing sample peak areas to standard calibration curves (Guo et al., 2022).

### 2.9 Statistical analysis

The data were analyzed using GraphPad Prism 9.0. The experiment data were expressed as mean ± standard deviation. Differences among multiple groups were assessed by one-way ANOVA followed by the Tukey–Kramer post-hoc test. The student’s t-test was used to analyze the comparison between the two groups. A value of *p* < 0.05 was considered statistically significant. Comparison with the NC group is denoted as #, with #*p* < 0.05, ##*p* < 0.01, and ###*p* < 0.001. Comparison with the MC group is denoted as *, with * *p* < 0.05, ** *p* < 0.01, and *** *p* < 0.001.

## 3 Results

### 3.1 GRG treatment improves constipation phenotype

**Figure 1:**
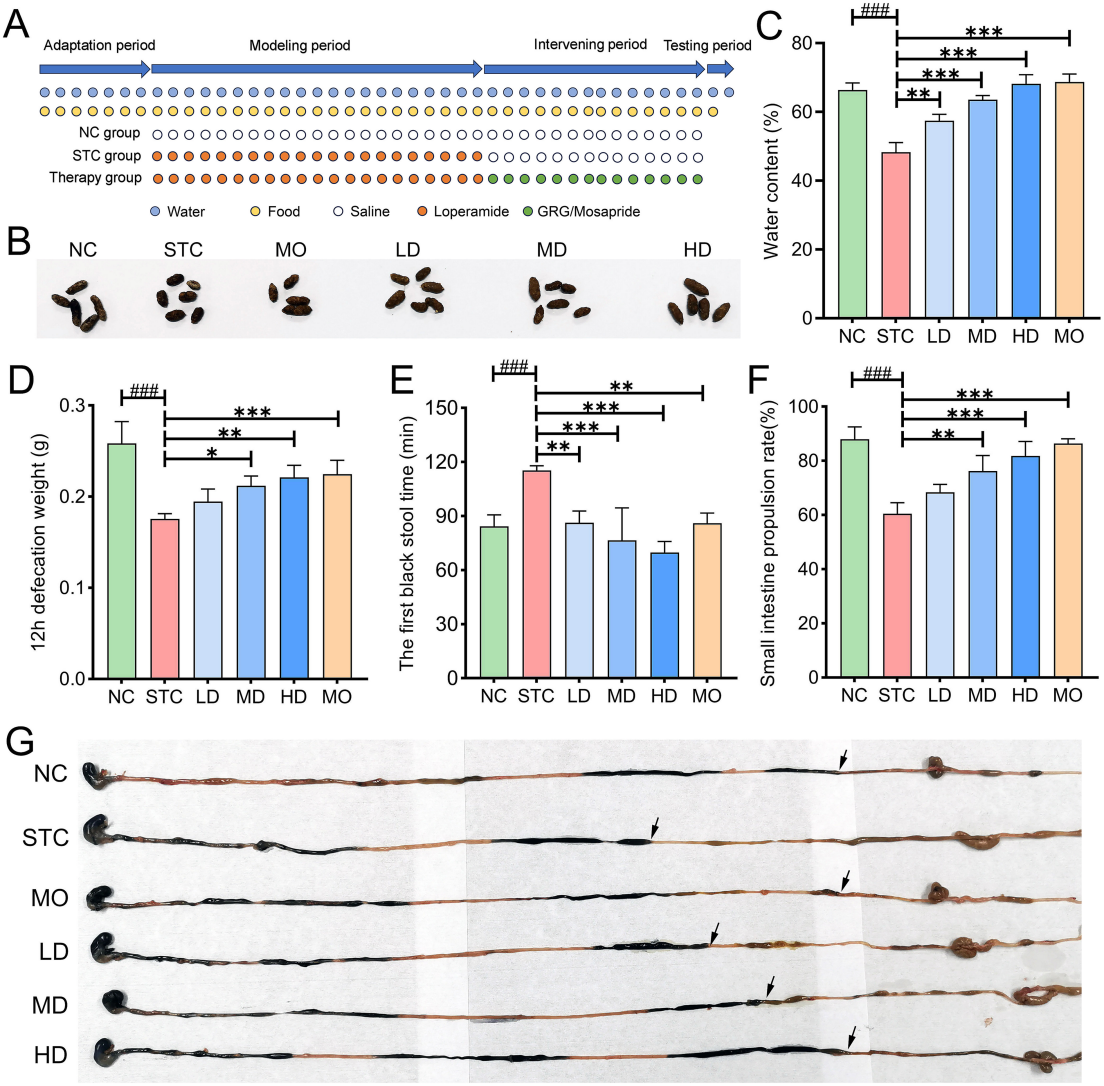
Effects of GRG on loperamide-induced constipation mice. **(A)** Experimental design. **(B)** The shape of collected feces. **(C)** Fecal water content. **(D)** The weight of 12 h feces. **(E)** The first black stool defecation time. **(F)** Gastrointestinal transit rate. **(G)** The intestinal propelling length of ink. Values are presented as mean ± SD, *n* = 4. ###*p* < 0.001, NC vs. STC. *p < 0.05, **p < 0.01, ***p < 0.001, LD, MD, HD and MO vs. STC.

The experimental work flow is outlined in Figure 1A. The therapeutic effects of GRG on constipated mice were evaluated following treatment. In terms of defecation phenotype, compared to the healthy group, model mice exhibited significantly smaller fecal pellets, reduced fecal moisture content, and a lower total fecal output over 12 h (*p* < 0.001). Treatment with GRG or mosapride significantly alleviated these symptoms (Figures 1B–D). In terms of gastrointestinal motility phenotype, loperamide administration markedly impaired gastrointestinal motility, evidenced by prolonged defecation time and a reduced intestinal transit rate (*p* < 0.001). Both GRG and mosapride significantly improved these motility impairments (Figures 1E–G). A dose-dependent effect was observed for GRG across all phenotypes. Notably, high-dose GRG exhibited therapeutic efficacy comparable to, or even exceeding, that of the positive control drug, mosapride.

### 3.2 GRG treatment repairs colon damage caused by constipation

**Figure 2:**
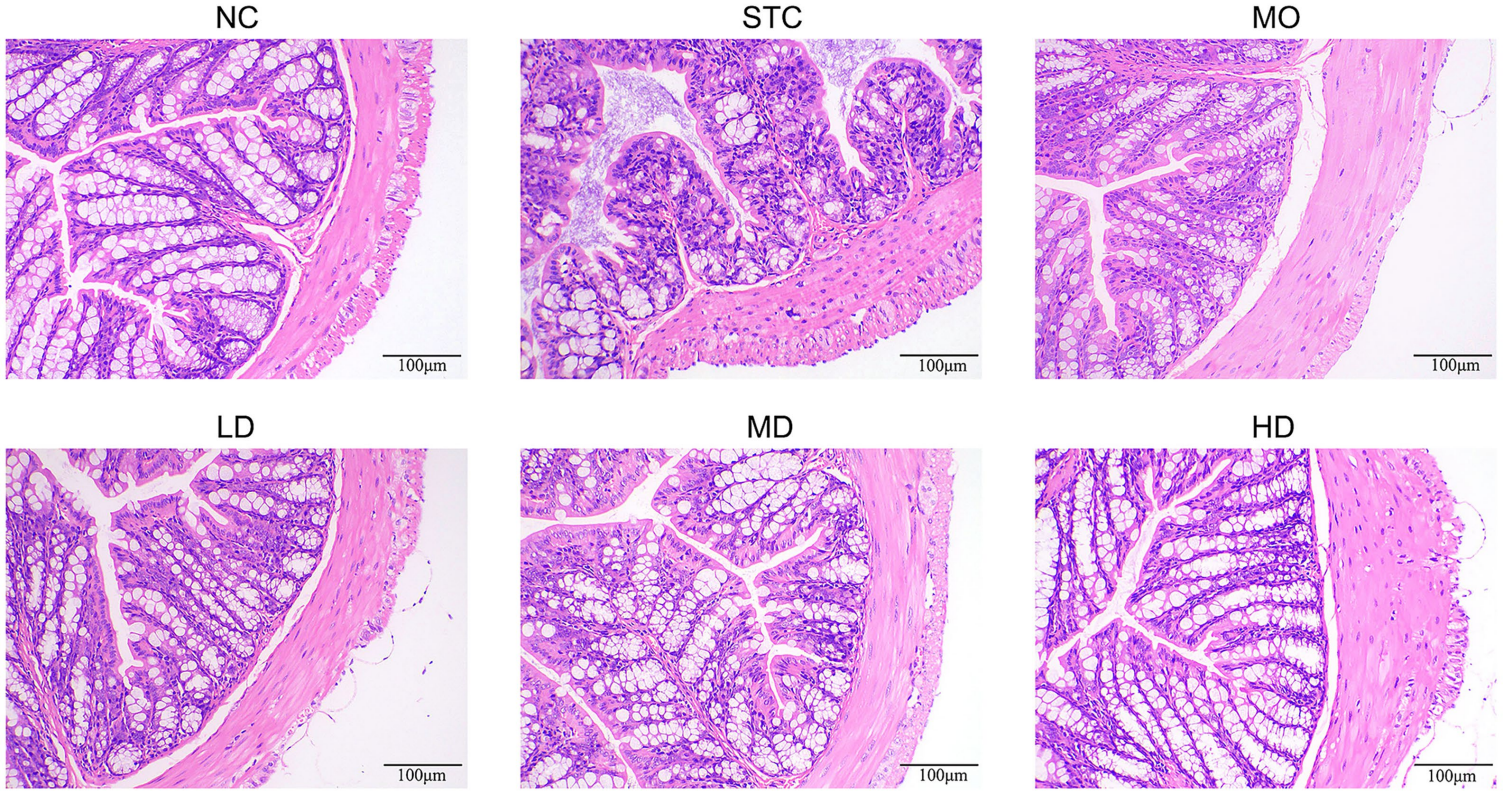
Effects of GRG on histology of colonic tissue.

H&E staining was employed to assess morphological changes in the colon (Figure 2). Constipation induced significant damage to the colonic tissue, characterized by irregular glandular arrangement, apparent reduction in goblet cells, and inflammatory cell infiltration. Treatment with GRG or mosapride effectively ameliorated these structural abnormalities, showing a trend towards restoration of a more normal colonic architecture with clearer glandular structure and a visible increase in goblet cells compared to the STC group.

### 3.3 GRG regulates motility-related hormone levels in colonic tissue and serum

**Figure 3:**
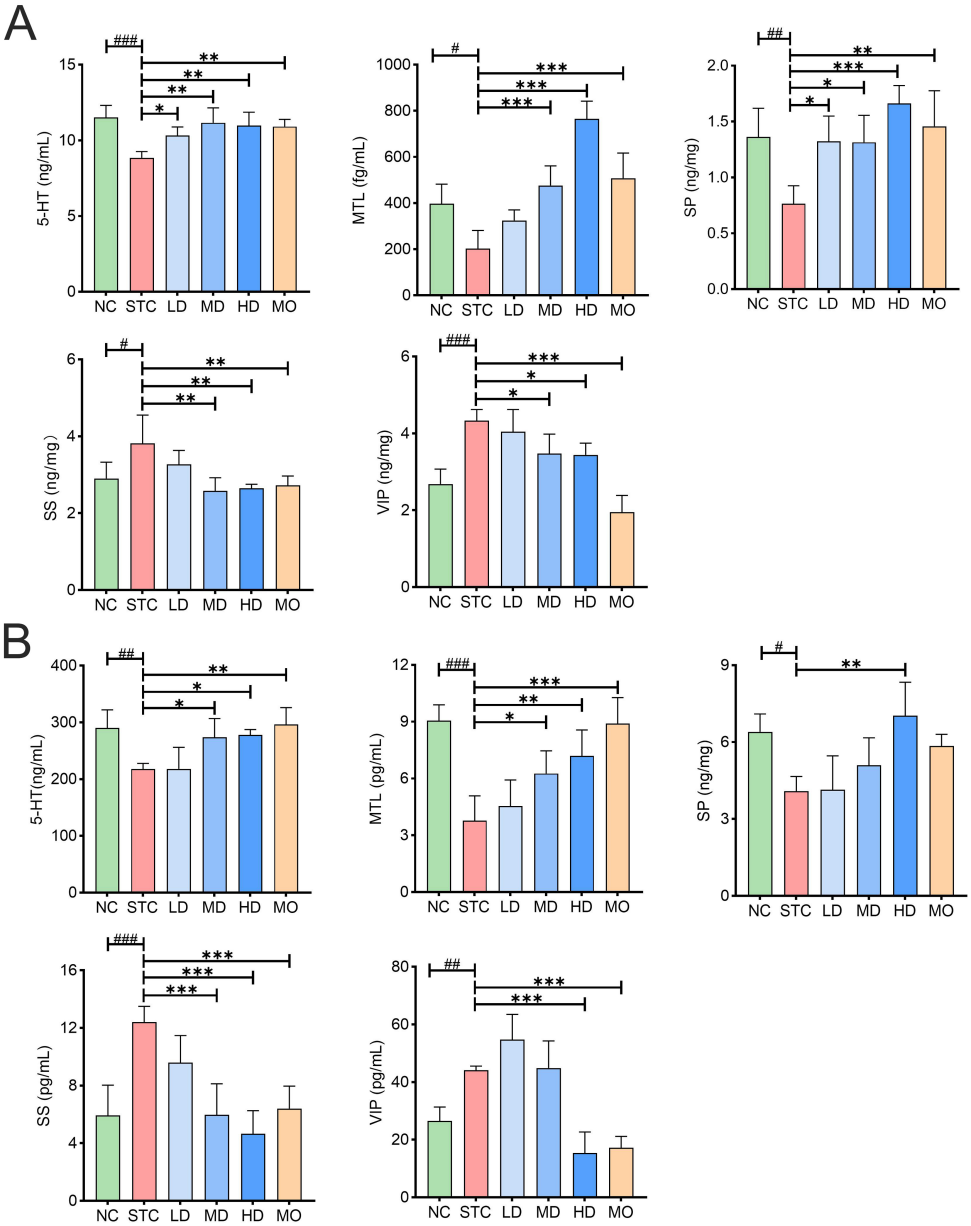
The effect of GRG treatment on the motility-related hormone in mice. **(A)** Hormone concentration in colon tissue. **(B)** Hormone concentration in serum. Values are presented as mean ± SD, *n* = 4. #*p* < 0.05; ##*p* < 0.01, ###*p* < 0.001, NC vs. STC. *p < 0.05, **p < 0.01, ***p < 0.001, LD, MD, HD and MO vs. STC.

Gastrointestinal motility is closely regulated by gut hormones. This study measured hormone levels in colonic tissue and serum using ELISA. As shown in Figure 3, loperamide significantly disrupted hormone levels, leading to decreased stimulatory hormones (5-HT, MTL, and SP) and increased inhibitory hormones (SS and VIP) in both colonic tissue and serum. Medium- and high-dose GRG treatments effectively restored the levels of all five hormones in colonic tissue (*p* < 0.05). The reparative effects of GRG on 5-HT, SP, and SS were comparable to those of mosapride, while high-dose GRG demonstrated a significantly stronger effect on MTL compared to mosapride. However, GRG’s impact on VIP was less pronounced than mosapride. A clear dose–response effect was observed for MTL and VIP. In serum, medium- and high-dose GRG treatments significantly restored 5-HT, MTL, and SS levels, while high-dose GRG also restored SP and VIP levels. High-dose GRG exhibited comparable effects to mosapride on serum 5-HT and VIP levels, with a more pronounced impact on SP and SS levels. Notably, the dose–response effect of GRG on hormone regulation was more pronounced in serum than in colonic tissue. These results indicate that GRG significantly regulates motility-related hormone secretion in both colonic tissue and serum, exhibiting a clear dose-dependent therapeutic effect.

### 3.4 GRG treatment improves the composition of the gut microbiota

**Figure 4:**
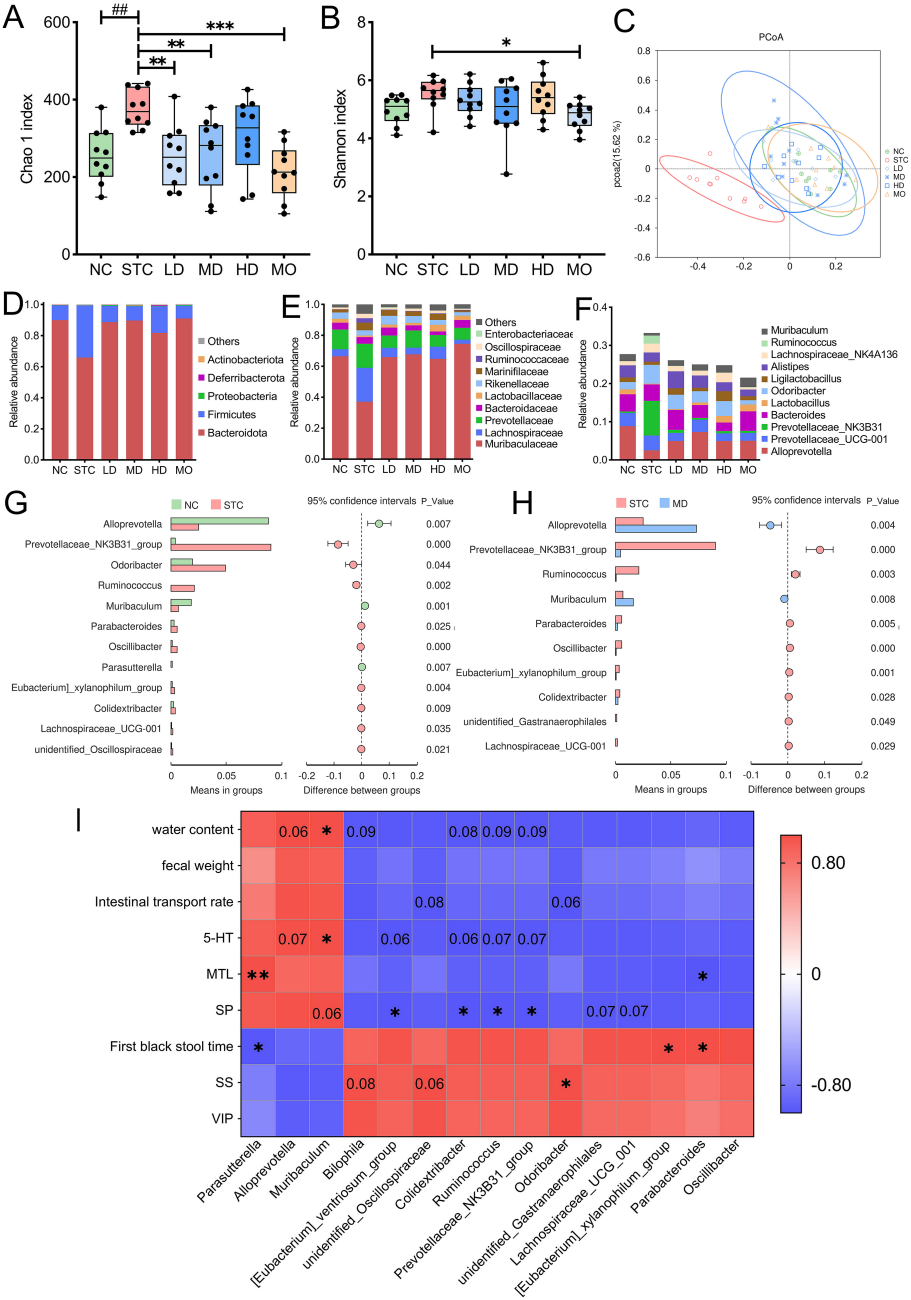
Effects of GRG treatment on fecal gut microbiota in mice. Alpha diversity measured by the Chao1 index **(A)** and Shannon index **(B)**, # *p* < 0.05; ## *p* < 0.01, ### *p* < 0.001, NC vs. STC. *p < 0.05, **p < 0.01, ***p < 0.001, LD, MD, HD and MO vs. STC. **(C)** Principal coordinate analysis (PCoA) based on weighted UniFrac distances. Taxonomic distribution of bacterial communities at the phylum level **(D)**, family level **(E)**, and genus level **(F)**. *T*-test analysis of differential bacterial genera at the genus level, NC vs. STC groups **(G)** and MD vs. STC groups **(H)**. The left panel shows the relative abundance ratios of different genera in the two samples, the middle panel indicates the proportion of functional abundance differences within the 95% confidence interval, and the right panel displays the *p*-value. **(I)** Pearson correlation analysis between key differential genera abundance and constipation-related indicators. Red and blue indicate positive and negative correlations between indicators, respectively. *p < 0.05; **p < 0.01. Values within the squares indicate results with a *p*-value between 0.05 and 0.1. Values are presented as mean ± SD, *n* = 10.

The effect of GRG on gut microbiota was evaluated by analyzing the microbiome of fecal samples. Alpha diversity, assessed using the Chao1 index, showed a notable increase in microbial abundance in the STC group relative to the NC group (*p* < 0.01) (Figures 4A,B). Mosapride treatment significantly reversed this change (*p* < 0.001). GRG treatment at low and medium doses significantly reduced microbial abundance (*p* < 0.01), while high-dose GRG also decreased abundance, though not significantly (*p* > 0.05). PCoA based on weighted UniFrac distances revealed significant differences in gut microbiota structure between the STC and NC groups. GRG treatment restored these structural changes (Figure 4C). Inter-group differences, analyzed using ANOSIM, confirmed significant dissimilarities between the microbiota of constipated mice and other groups (*R* > 0.72, *p* < 0.01) (Supplementary Table S2).

To reveal the specific impact of GRG on the microbiome, the composition of the dominant microbiota was analyzed. At the phylum level, Bacteroidota was the dominant phylum across all groups, accounting for 89.9, 65.9, 88.7, 89.7, 81.8, and 91.0% in the NC, STC, LD, MD, HD, and MO groups, respectively, followed by Firmicutes, which showed relative abundances of 9.6, 33.5, 10.5, 9.3, 17.1, and 8.3%. Significant differences in the abundances of Bacteroidota and Firmicutes were noted between the NC and STC groups (*p* < 0.01), with all treatments effectively reversing these changes (*p* < 0.01) (Figure 4D).

At the family level (Figure 4E), Muribaculaceae was the most abundant family in all groups, accounting for 66.5, 37.2, 65.9, 67.7, 64.8, and 74.6% in the NC, STC, LD, MD, HD, and MO groups, respectively. The STC group showed a notable reduction in Muribaculaceae and an elevation in Lachnospiraceae abundance compared to other groups (*p* < 0.05).

At the genus level (Figure 4F), *Alloprevotella*, *Prevotellaceae_UCG-001*, *Bacteroides*, and *Alistipes* were dominant across all groups. Differential analysis using a *t*-test revealed significant differences between the NC group, the MD group, and the STC group. Compared to the STC group, the NC and MD groups showed significant enrichment of *Alloprevotella* and *Muribaculum* (Figures 4G–H). Conversely, genera such as *Prevotellaceae_NK3B31_group*, *Ruminococcus*, *Lachnospiraceae_UCG-001*, *Parabacteroides*, *Eubacterium_xylanophilum_group*, and *Colidextribacter* were significantly enriched in the STC group. Additionally, *Odoribacter* was predominantly enriched in the STC group, while *Parasutterella* was distinctly reduced compared to the NC group. To further investigate the role of these genera in GRG-mediated constipation treatment, Pearson correlation analysis was performed between bacterial abundance and constipation-related indicators. As shown in Figure 4I, bacteria enriched in the NC and MD groups-such as *Alloprevotella*, *Parasutterella*, and *Muribaculum-*were positively correlated with defecation-promoting indicators and excitatory hormones, such as MTL, 5-HT, and SP. These genera were also negatively correlated with inhibitory hormones and defecation time, suggesting their role in promoting defecation. In contrast, bacteria enriched in the STC group-such as *Prevotellaceae_NK3B31_group*, *Ruminococcus*, and *Colidextribacter*-showed the opposite correlations, indicating a potential link to constipation. Notably, *Parasutterella* abundance was positively correlated with MTL levels (*p* < 0.01), while *Muribaculum* abundance was positively correlated with 5-HT and SP levels. In contrast, *[Eubacterium]_xylanophilum_group*, *Colidextribacter*, *Ruminococcus*, and *Prevotellaceae_NK3B31_group* showed negative correlations with 5-HT and SP concentrations (*p* < 0.1). *Parabacteroides* was negatively correlated with MTL levels (*p* < 0.05).

### 3.5 GRG therapy restores the metabolic function of gut microbiota

**Figure 5:**
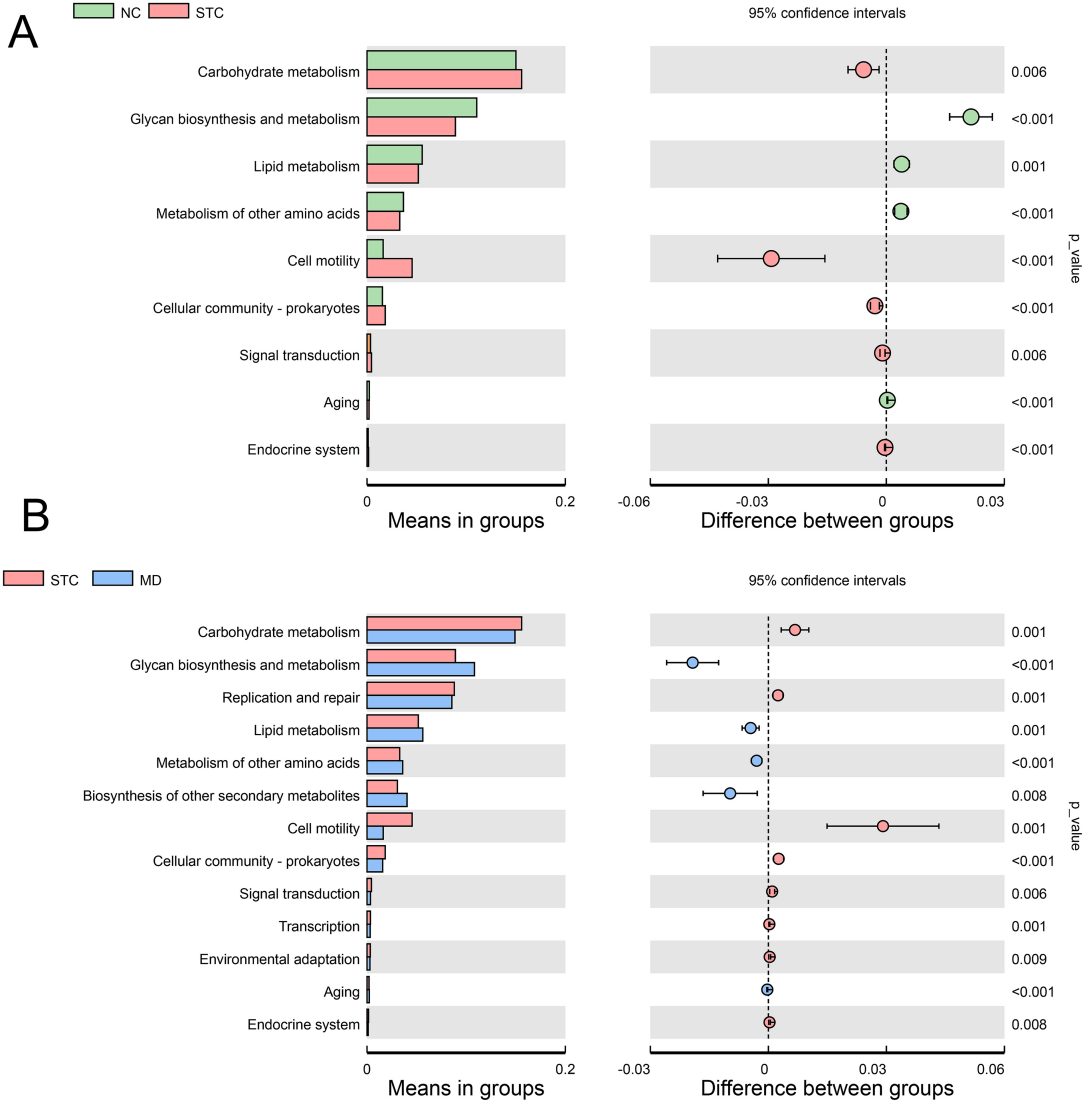
GRG therapy improved the metabolic function of gut microbiota. Picrust 2 predicted significantly changed KEGG pathways between NC vs. SC groups **(A)** and MD vs. STC groups **(B)**. The figure on the left shows the abundance ratio of different functions in two groups. In the middle is the proportion of differences in functional abundance within the 95% confidence interval. The right is the *p*-value.

To explore the functional changes in gut microbiota associated with GRG treatment, PICRUSt2 was employed to predict bacterial metabolic functional profiles. KEGG pathway enrichment analysis identified 13 pathways with significant alterations (Figure 5). In the STC group, pathways related to carbohydrate metabolism, cell motility, and cellular community–prokaryotes were significantly upregulated, while metabolic pathways involved in glycan biosynthesis and metabolism, lipid metabolism, and metabolism of other amino acids were markedly suppressed (*p* < 0.01). After GRG treatment, these functional abnormalities were significantly restored (*p* < 0.01). Additionally, GRG therapy notably enhanced the biosynthesis of other secondary metabolites. These findings indicated that GRG treatment effectively regulated the metabolic functions of gut microbiota in constipated mice.

### 3.6 GRG treatment enhances SCFAs content in feces

**Figure 6:**
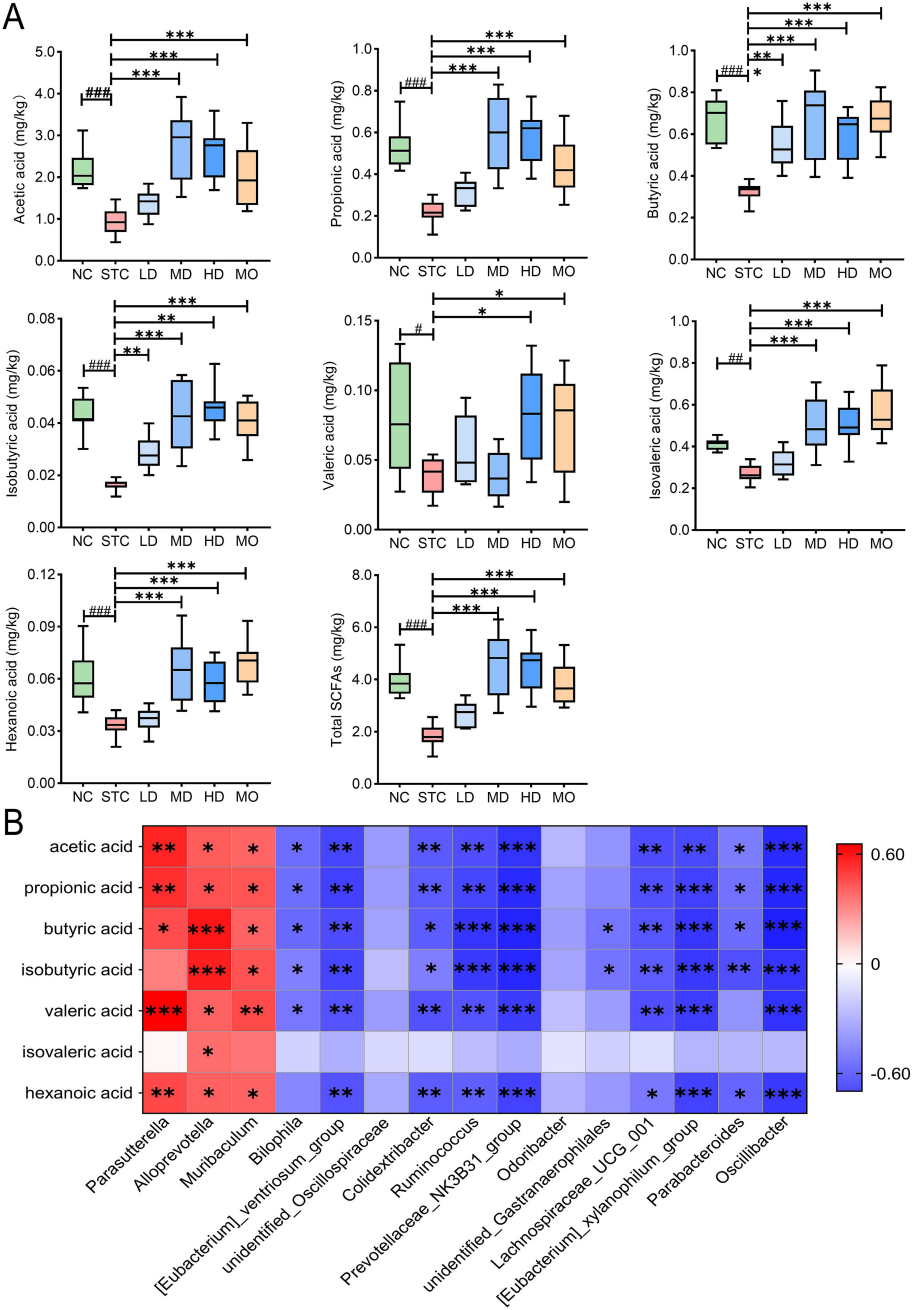
GRG treatment affects fecal SCFA contents. **(A)** Fecal SCFA contents in different groups. Total SCFAs stand for the total content of all the seven SCFAs. #*p* < 0.05; ##*p* < 0.01, ###*p* < 0.001, NC vs. STC. *p < 0.05, **p < 0.01, ***p < 0.001, LD, MD, HD and MO vs. STC. **(B)** Correlation analysis between differential genus microbiota and SCFA abundance. Correlation analysis was performed by the Pearson method. The red and blue colors stand for the positive and negative correlation between the two indicators, respectively. Values are presented as mean ± SD, *n* = 10. *p < 0.05; **p < 0.01; ***p < 0.001.

Short-chain fatty acids are essential secondary metabolites of the gut microbiota. The fecal levels of SCFAs were measured. As shown in Figure 6A, the total SCFAs content in the feces of constipated mice was notably reduced compared to the healthy group (*p* < 0.001). Specifically, the levels of acetic acid, propionic acid, butyric acid, isobutyric acid, and caproic acid were markedly decreased (*p* < 0.001), while valeric acid and isovaleric acid showed a declining trend (*p* < 0.05). Treatment with medium and high doses of GRG significantly increased the levels of all SCFAs except valeric acid (*p* < 0.01). Low-dose GRG treatment only significantly increased butyrate and isobutyrate levels (*p* < 0.01).

Pearson correlation analysis was implemented to examine the relationship between SCFAs level and the abundance of key bacterial genera. As illustrated in Figure 6B, the abundance of *Parasutterella*, *Alloprevotella*, and *Muribaculum* was positively correlated with SCFAs content, indicating their potential role in SCFAs synthesis. Notably, *Parasutterella* showed a significant positive correlation with acetate, propionate, valerate, and caproate (*p* < 0.01), while *Alloprevotella* was significantly correlated with butyrate and isobutyrate levels (*p* < 0.01). In contrast, genera such as *Prevotellaceae_NK3B31_group*, *Oscillibacter*, *[Eubacterium]_xylanophilum_group*, *[Eubacterium]_ventriosum_group*, *Ruminococcus*, *Lachnospiraceae_UCG-001*, and *Colidextribacter* exhibited a significant negative correlation with the content of most SCFAs (*p* < 0.01).

## 4 Discussion

### 4.1 TCM GRG exhibits substantial therapeutic efficacy in constipation management

Traditional Chinese medicine has been used for thousands of years to treat diseases in Asia, emerging clinical and preclinical evidence underscores the therapeutic potential of TCM formulations in constipation management. Clinically, TCM formulations, such as Shouhui Tongbian Capsules, has demonstrated significant therapeutic effects, improving clinical outcomes such as spontaneous bowel movement frequency, stool consistency, the sensation of complete evacuation, and overall quality of life, with durability surpassing conventional therapies (Guo et al., 2023). Preclinical investigations utilizing animal models of constipation further corroborate these findings and highlight its potential impact on various physiological and pathological factors, including gut motility hormones, mucosal barriers, aquaporins, inflammation, gut microbiota, and microbial metabolites (Tuohongerbieke et al., 2024).

Previous research revealed that GRG effectively alleviated morphine-induced STC symptoms in mice by downregulating colonic aquaporin 4 (AQP4) expression and restoring the SCF/c-kit pathway (Sun et al., 2020). In the current investigation, GRG administration significantly ameliorated defecation parameters in loperamide-induced constipated mice, evidenced by elevated fecal water content, augmented fecal output, enhanced colonic motility, and histological restoration of intestinal integrity. Notably, this study highlights the therapeutic mechanism of GRG in the treatment of constipation, focusing on its role in restoring the gut microbiota.

### 4.2 Gut microbiota as a pivotal mediator in constipation pathogenesis and TCM therapeutic mechanisms

The intricate interplay between gut microbiota and human health has been widely recognized in recent decades (Lynch and Pedersen, 2016). As the largest microbial reservoir in humans, the intestinal ecosystem maintains dynamic equilibrium through symbiotic relationships with its microbial inhabitants (Ohkusa et al., 2019). Emerging evidence positions gut dysbiosis as a hallmark feature of STC. Clinical researches has identified distinct microbial signatures in STC patients (Guo et al., 2020; Han K. et al., 2024). Furthermore, transferring the gut microbiota from constipated patients to mice has been shown to induce constipation-like symptoms, causally linking microbial dysbiosis to constipation pathogenesis. Gut microbiota restoration interventions, including probiotics and fecal microbiota transplantation, have shown remarkable efficacy in alleviating constipation (Ma et al., 2023). Dietary changes and prebiotics treatments for target constipation, have been shown to restore gut microbiota (Lai et al., 2023). These findings underscore the microbiota’s dual role as both a disease modulator and therapeutic target. Restoring the balance of the microbiota not only alleviates symptoms, but also serves as a key indicator of treatment efficacy (Schupack et al., 2022; Sorbara and Pamer, 2022).

Metabolic disorders such as constipation often have multifactorial causes. TCM formulas contain various bioactive components such as polysaccharides, polypeptides, and alkaloids. These components enable TCM to exert therapeutic effects via multi-target and multi-pathway mechanisms, providing a holistic treatment strategy for constipation management. Numerous studies highlight TCM exert multimodal therapeutic effects by simultaneously restoring microbial ecology, enhancing beneficial metabolites, and repairing mucosal integrity (Zhan et al., 2022; Gou et al., 2023). Central to this paradigm is the bi-directional crosstalk between TCM phytochemicals and gut microbiota (Cheng et al., 2023). For instance, polysaccharides in TCM formulas can promote the growth of beneficial gut bacteria, restore intestinal homeostasis, while also being metabolized by microorganisms to produce active metabolites such as SCFAs (Yue et al., 2022). Microbiota-derived SCFAs play a multifaceted role by serving as an energy source for gastrointestinal neurons, suppressing inflammation, stimulating the secretion of gastrointestinal regulatory peptides, enhancing gastrointestinal motility, and ultimately promoting defecation (Martin-Gallausiaux et al., 2021). Given the crucial role of gut microbiota in mediating the therapeutic effect of traditional Chinese medicine, gut microbiota restoration has become an important direction for studying the mechanisms of traditional Chinese medicine in treating metabolic diseases such as constipation (Zhang et al., 2021; Wang et al., 2024).

### 4.3 GRG regulates gut microbiota and metabolites to treat constipation

The strong correlations observed between the enrichment of beneficial bacteria (e.g., Alloprevotella and Muribaculum), the rise in SCFAs, and the normalization of motility-related hormones suggest a potential mechanistic cascade whereby GRG modulates the gut microbiome, leading to changes in microbial metabolites that in turn influence host hormone secretion and gut function. Our results strongly support this hypothesis and provide a framework for future mechanistic investigations. GRG treatment effectively reversed constipation-induced gut microbiota dysbiosis, characterized by increased *Alloprevotella* and *Muribaculum* abundance and reduced *Ruminococcus*, *Prevotellaceae_NK3B31_group*, *Lachnospiraceae_UCG-001*, *Parabacteroides*, and *Colidextribacter*. The abundance of key microbial taxa strongly correlated with constipation-related phenotypes, suggesting their important roles in GRG’s therapeutic effects for STC. Among these key bacteria, *Alloprevotella* was considered a producer of SCFAs (Han B. et al., 2024). In this study, *Alloprevotella* abundance positively correlated with butyrate and isobutyrate levels (*p* < 0.001). Similarly, reduced *Muribaculum* abundance has been observed in constipated mice. *Prevotellaceae_NK3B31_group* presents a more complex relationship with constipation. Yang et al. found it significantly enriched in constipated rats and positively correlated with inflammation markers (Yang et al., 2021). However, other studies reported a decrease in *Prevotellaceae_NK3B31_group* abundance in constipated group (Tuohongerbieke et al., 2024). In this study, the *Prevotellaceae_NK3B31_group* was significantly enriched in constipated mice and negatively correlated with SCFA levels (*p* < 0.001). Clinical research has also identified increased *Ruminococcus* abundance in elderly patients with constipation (Guo et al., 2020). TCM treatments have been shown to significantly reduce *Ruminococcus* levels in constipated rats, leading to changes in metabolite levels and therapeutic benefits (Li et al., 2023). However, variations in key microbial taxa, such as *Alloprevotella*, *Prevotellaceae_NK3B31_group*, and *Ruminococcus*, across different constipation studies highlight the complexity of their relationship with constipation. Further research is needed to clarify these associations and better understand their roles in TCM’s treatment mechanisms.

The gut microbiota produced secondary metabolites playing essential roles in regulating host physiology (Krautkramer et al., 2021). This study identified significant changes in the metabolic functions of the gut microbiota in constipated mice. GRG treatment enhanced microbiota functions related to glycan biosynthesis and metabolism, lipid metabolism, metabolism of other amino acids, and biosynthesis of secondary metabolites, highlighting the critical role of microbial metabolites in the therapeutic effects of GRG on constipation. Additionally, GRG treatment significantly increased SCFAs level in constipated mice, consistent with the observed improvement in glycan biosynthesis and metabolism. In this study, GRG treatment restored the abundance of SCFA-producing bacteria such as *Alloprevotella*, which was positively correlated with butyrate and other SCFAs level. Research suggested that TCM could regulate gut motility related hormones by improving gut microbiota (Gao et al., 2022). Besides, GRG treatment significantly increased the levels of stimulating hormones such as 5-HT and SP, which was positively correlated with the increased abundance of bacteria such as *Muribaculum* and *Alloprevotella*. Both 5-HT and SP are critical hormones associated with colonic motility. SP enhances smooth muscle contraction, stimulates gastrointestinal peristalsis, and promotes intestinal secretion (Wang et al., 2023a), while 5-HT facilitates intestinal secretion and motility (Israelyan et al., 2019). Notably, this observation aligns with prior demonstrations that *Alloprevotella*-derived butyrate potentiates enterochromaffin cell activity, thereby amplifying serotonin signaling to enhance intestinal motility (Vincent et al., 2018; Han et al., 2024a). Furthermore, the potential mechanism by which SCFAs like butyrate influence motility may extend beyond hormonal secretion to direct effects on the enteric nervous system (ENS). Research has shown that chronic constipation is associated with ENS dysfunction, including neurochemical abnormalities, loss of nerve fibers, and a reduction in ICC (Bassotti et al., 2011). Notably, impaired butyrate metabolism has been implicated in causing ENS damage, which may be a critical factor in functional constipation (Wang et al., 2023b). Given that GRG treatment in our study significantly increased butyrate-producing bacteria (*Alloprevotella*) and fecal butyrate levels, it is plausible that the amelioration of constipation symptoms was partly mediated through the restoration of butyrate-supported ENS function. These findings suggested that the gut microbiota and its metabolites play an integral role in the mechanisms underlying the efficacy of GRG in treating constipation.

However, this study has several limitations that should be considered. The experiments were conducted exclusively in male mice, which precludes exploration of potential sex-specific differences in GRG’s therapeutic effects. And functional predictions via PICRUSt2 depend on the completeness of reference databases and phylogenetic inference; horizontal gene transfer, uncultured microbes, and unannotated genomes may bias pathway predictions. Additionally, although H&E staining indicated that GRG ameliorated histopathological damage, the assessment remained morphological. A more quantitative and mechanistic assessment of intestinal barrier repair, using mucin-specific stains (e.g., PAS) or immunohistochemistry for tight junction proteins (e.g., ZO-1, occludin) would offer deeper mechanistic insight into intestinal barrier repair. Moreover, while GRG modulated overall gut microbiota structure, its key active ingredients and their specific roles were not elucidated. Finally, although compelling systemic correlations were observed, the spatial and cellular mechanisms within the gut wall, such as involvement of specific enteroendocrine cells or enteric neurons, remain unclear.

These limitations highlight the need for future studies incorporating both sexes and employing metagenomic or metabolomic profiling to resolve strain-specific functions and validate microbial metabolite–host interactions. Furthermore, advanced metabolomics should be applied to characterize the chemical profile of GRG, identify its key pharmacological components, and evaluate their effects on specific bacterial groups, SCFA production, and intestinal motility. In addition, future studies utilizing techniques such as immunohistochemistry for neuronal markers (e.g., PGP9.5) and key hormones (e.g., 5-HT) are crucial to visualize and confirm these interactions directly at the cellular level, moving from correlation to definitive causation. Such approaches would strengthen causal interpretations of GRG’s microbiota-mediated mechanisms in constipation management.

## 5 Conclusion

GRG treatment significantly improved defecation phenotypes and increased intestinal motility in loperamide-induced constipated mice. It also effectively regulated the levels of motility-related hormones levels. The gut microbiota appears to play a key role in the therapeutic effects of GRG. By modulating gut microbiota composition, restoring SCFAs level, and restoring intestinal motility hormones, GRG offers a promising approach for the treatment of constipation.

## Data Availability

The datasets presented in this study are publicly available. This data can be found at: https://www.ncbi.nlm.nih.gov/sra, accession numbers PRJNA1203378 and PRJNA1119701.

## References

[ref1] Barberio B. Judge C. Savarino E. V. Ford A. C. (2021). Global prevalence of functional constipation according to the Rome criteria: a systematic review and meta-analysis. Lancet Gastroenterol. Hepatol. 6, 638–648. doi: 10.1016/s2468-1253(21)00111-4, PMID: 34090581

[ref2] Bassotti G. Villanacci V. Rostami N. M. (2011). Chronic constipation: no more idiopathic, but a true neuropathological entity. Gastroenterol. Hepatol. Bed Bench 4, 109–115. 24834167 PMC4017417

[ref3] Bharucha A. E. Lacy B. E. (2020). Mechanisms, evaluation, and management of chronic constipation. Gastroenterology 158, 1232–1249.e3. doi: 10.1053/j.gastro.2019.12.034, PMID: 31945360 PMC7573977

[ref4] Bhattarai Y. Williams B. B. Battaglioli E. J. Whitaker W. R. Till L. Grover M.. (2018). Gut microbiota-produced tryptamine activates an epithelial G-protein-coupled receptor to increase colonic secretion. Cell Host Microbe 23, 775–785.e5. doi: 10.1016/j.chom.2018.05.004, PMID: 29902441 PMC6055526

[ref5] Cheng H. Zhang D. Wu J. Liu J. Zhou Y. Tan Y.. (2023). Interactions between gut microbiota and polyphenols: a mechanistic and metabolomic review. Phytomedicine 119:154979. doi: 10.1016/j.phymed.2023.154979, PMID: 37552899

[ref6] Fan Y. Pedersen O. (2021). Gut microbiota in human metabolic health and disease. Nat. Rev. Microbiol. 19, 55–71. doi: 10.1038/s41579-020-0433-9, PMID: 32887946

[ref7] Gao X. Hu Y. Tao Y. Liu S. Chen H. Li J.. (2022). *Cymbopogon citratus* (DC.) Stapf aqueous extract ameliorates loperamide-induced constipation in mice by promoting gastrointestinal motility and regulating the gut microbiota. Front. Microbiol. 13:1017804. doi: 10.3389/fmicb.2022.1017804, PMID: 36267178 PMC9578511

[ref8] Gou H. Su H. Liu D. Wong C. C. Shang H. Fang Y.. (2023). Traditional medicine Pien Tze Huang suppresses colorectal tumorigenesis through restoring gut microbiota and metabolites. Gastroenterology 165, 1404–1419. doi: 10.1053/j.gastro.2023.08.052, PMID: 37704113

[ref9] Guo X. Li R. Huang N. Zhang T. Li J. Gong L.. (2023). Efficacy and safety of Shouhui Tongbian capsules in the treatment of constipation: a systematic review and meta-analysis. Phytomedicine 108:154541. doi: 10.1016/j.phymed.2022.154541, PMID: 36375236

[ref10] Guo Y. Song L. Huang Y. Li X. Xiao Y. Wang Z.. (2022). *Latilactobacillus sakei* Furu2019 and stachyose as probiotics, prebiotics, and synbiotics alleviate constipation in mice. Front. Nutr. 9:1039403. doi: 10.3389/fnut.2022.1039403, PMID: 36687730 PMC9849682

[ref11] Guo M. Yao J. Yang F. Liu W. Bai H. Ma J.. (2020). The composition of intestinal microbiota and its association with functional constipation of the elderly patients. Future Microbiol. 15, 163–175. doi: 10.2217/fmb-2019-0283, PMID: 32079430

[ref12] Han K. Kuo B. Khalili H. Staller K. (2024). Metagenomics analysis reveals unique gut microbiota signature of slow-transit constipation. Clin. Transl. Gastroenterol. 15:e1. doi: 10.14309/ctg.0000000000000766, PMID: 39225513 PMC11500773

[ref13] Han B. Shi L. Bao M. Y. Yu F. L. Zhang Y. Lu X. Y.. (2024). Dietary ellagic acid therapy for CNS autoimmunity: targeting on *Alloprevotella rava* and propionate metabolism. Microbiome 12:114. doi: 10.1186/s40168-024-01819-8, PMID: 38915127 PMC11194905

[ref14] Harris L. A. Chang C. H. (2022). Burden of constipation: looking beyond bowel movements. Am. J. Gastroenterol. 117, S2–s5. doi: 10.14309/ajg.0000000000001708, PMID: 35354769

[ref15] Israelyan N. Del Colle A. Li Z. Park Y. Xing A. Jacobsen J. P. R.. (2019). Effects of serotonin and slow-release 5-Hydroxytryptophan on gastrointestinal motility in a mouse model of depression. Gastroenterology 157, 507–521.e4. doi: 10.1053/j.gastro.2019.04.022, PMID: 31071306 PMC6650329

[ref16] Jia A. Niu X. Zhang M. Liu X. Cui T. Liu C.. (2024). Integrated microbiomics and metabolomics analysis reveals the influence of gut microbiota on the growth and metabolism of sea cucumber seedlings. J. Appl. Microbiol. 135:lxae006. doi: 10.1093/jambio/lxae006 38211975

[ref17] Krautkramer K. A. Fan J. Bäckhed F. (2021). Gut microbial metabolites as multi-kingdom intermediates. Nat. Rev. Microbiol. 19, 77–94. doi: 10.1038/s41579-020-0438-4, PMID: 32968241

[ref18] Lai H. Li Y. He Y. Chen F. Mi B. Li J.. (2023). Effects of dietary fibers or probiotics on functional constipation symptoms and roles of gut microbiota: a double-blinded randomized placebo trial. Gut Microbes 15:2197837. doi: 10.1080/19490976.2023.2197837, PMID: 37078654 PMC10120550

[ref19] Li X. Wang X. Wang Z. Guan J. (2023). Baizhu-Baishao herb pair ameliorates functional constipation and intestinal microflora disorder in rats. Animal Model Exp Med 6, 598–608. doi: 10.1002/ame2.12351, PMID: 37859536 PMC10757208

[ref20] Liu B. Zhang Z. Liu X. Hu W. Wu W. (2023). Gastrointestinal fermentable polysaccharide is beneficial in alleviating loperamide-induced constipation in mice. Nutrients 15:4364. doi: 10.3390/nu15204364, PMID: 37892439 PMC10610129

[ref21] Lynch S. V. Pedersen O. (2016). The human intestinal microbiome in health and disease. N. Engl. J. Med. 375, 2369–2379. doi: 10.1056/NEJMra1600266, PMID: 27974040

[ref22] Ma T. Yang N. Xie Y. Li Y. Xiao Q. Li Q.. (2023). Effect of the probiotic strain, *Lactiplantibacillus plantarum* P9, on chronic constipation: a randomized, double-blind, placebo-controlled study. Pharmacol. Res. 191:106755. doi: 10.1016/j.phrs.2023.106755, PMID: 37019193

[ref23] Martens E. C. Neumann M. Desai M. S. (2018). Interactions of commensal and pathogenic microorganisms with the intestinal mucosal barrier. Nat. Rev. Microbiol. 16, 457–470. doi: 10.1038/s41579-018-0036-x, PMID: 29904082

[ref24] Martin-Gallausiaux C. Marinelli L. Blottière H. M. Larraufie P. Lapaque N. (2021). SCFA: mechanisms and functional importance in the gut. Proc. Nutr. Soc. 80, 37–49. doi: 10.1017/s0029665120006916, PMID: 32238208

[ref25] Ohkusa T. Koido S. Nishikawa Y. Sato N. (2019). Gut microbiota and chronic constipation: a review and update. Front Med (Lausanne) 6:19. doi: 10.3389/fmed.2019.00019, PMID: 30809523 PMC6379309

[ref26] Pannemans J. Masuy I. Tack J. (2020). Functional constipation: individualising assessment and treatment. Drugs 80, 947–963. doi: 10.1007/s40265-020-01305-z, PMID: 32451924

[ref27] Schupack D. A. Mars R. A. T. Voelker D. H. Abeykoon J. P. Kashyap P. C. (2022). The promise of the gut microbiome as part of individualized treatment strategies. Nat. Rev. Gastroenterol. Hepatol. 19, 7–25. doi: 10.1038/s41575-021-00499-1, PMID: 34453142 PMC8712374

[ref28] Shin A. Preidis G. A. Shulman R. Kashyap P. C. (2019). The gut microbiome in adult and pediatric functional gastrointestinal disorders. Clin. Gastroenterol. Hepatol. 17, 256–274. doi: 10.1016/j.cgh.2018.08.054, PMID: 30153517 PMC6314902

[ref29] Song E. M. Lee H. J. Jung K. W. Kim M. J. Hwang S. W. Park S. H.. (2021). Long-term risks of Parkinson's disease, surgery, and colorectal cancer in patients with slow-transit constipation. Clin. Gastroenterol. Hepatol. 19, 2577–2586.e6. doi: 10.1016/j.cgh.2020.08.059, PMID: 32882425

[ref30] Sorbara M. T. Pamer E. G. (2022). Microbiome-based therapeutics. Nat. Rev. Microbiol. 20, 365–380. doi: 10.1038/s41579-021-00667-9, PMID: 34992261

[ref31] Sumida K. Molnar M. Z. Potukuchi P. K. Thomas F. Lu J. L. Yamagata K.. (2019). Constipation and risk of death and cardiovascular events. Atherosclerosis 281, 114–120. doi: 10.1016/j.atherosclerosis.2018.12.021, PMID: 30658186 PMC6399019

[ref32] Sun Y. Yan C. Jin S. Shi C. Zhao J. Li G. (2020). Curative effect and mechanism of Guiren Runchang granules on morphine-induced slow transit constipation in mice. Evid. Based Complement. Alternat. Med. 2020:5493192. doi: 10.1155/2020/5493192, PMID: 33029167 PMC7530485

[ref33] Tuohongerbieke A. Wang H. Wu J. Wang Z. Dong T. Huang Y.. (2024). Xiao Cheng Qi decoction, an ancient Chinese herbal mixture, relieves loperamide-induced slow-transit constipation in mice: an action mediated by gut microbiota. Pharmaceuticals (Basel) 17:153. doi: 10.3390/ph17020153, PMID: 38399368 PMC10892578

[ref34] Vicentini F. A. Keenan C. M. Wallace L. E. Woods C. Cavin J. B. Flockton A. R.. (2021). Intestinal microbiota shapes gut physiology and regulates enteric neurons and glia. Microbiome 9:210. doi: 10.1186/s40168-021-01165-z, PMID: 34702353 PMC8549243

[ref35] Vincent A. D. Wang X. Y. Parsons S. P. Khan W. I. Huizinga J. D. (2018). Abnormal absorptive colonic motor activity in germ-free mice is rectified by butyrate, an effect possibly mediated by mucosal serotonin. Am. J. Physiol. Gastrointest. Liver Physiol. 315, G896–g907. doi: 10.1152/ajpgi.00237.2017, PMID: 30095295

[ref36] Vriesman M. H. Koppen I. J. N. Camilleri M. Di Lorenzo C. Benninga M. A. (2020). Management of functional constipation in children and adults. Nat. Rev. Gastroenterol. Hepatol. 17, 21–39. doi: 10.1038/s41575-019-0222-y, PMID: 31690829

[ref37] Wang L. Lv W. Q. Yang J. T. Lin X. Liu H. M. Tan H.-J.. (2023b). Enteric nervous system damage caused by abnormal intestinal butyrate metabolism may lead to functional constipation. Front. Microbiol. 14:1117905. doi: 10.3389/fmicb.2023.1117905, PMID: 37228368 PMC10203953

[ref38] Wang K. Qiu H. Chen F. Cai P. Qi F. (2024). Considering traditional Chinese medicine as adjunct therapy in the management of chronic constipation by regulating intestinal flora. Biosci. Trends 18, 127–140. doi: 10.5582/bst.2024.01036, PMID: 38522913

[ref39] Wang L. Wu F. Hong Y. Shen L. Zhao L. Lin X. (2022). Research progress in the treatment of slow transit constipation by traditional Chinese medicine. J. Ethnopharmacol. 290:115075. doi: 10.1016/j.jep.2022.115075, PMID: 35134487

[ref40] Wang L. Xie S. Jiang X. Xu C. Wang Y. Feng J.. (2023a). Therapeutic effects of *Bombax ceiba* flower aqueous extracts against loperamide-induced constipation in mice. Pharm. Biol. 61, 125–134. doi: 10.1080/13880209.2022.2157841, PMID: 36582187 PMC9809371

[ref41] Yang Z. Ye S. Xu Z. Su H. Tian X. Han B.. (2021). Dietary synbiotic ameliorates constipation through the modulation of gut microbiota and its metabolic function. Food Res. Int. 147:110569. doi: 10.1016/j.foodres.2021.110569, PMID: 34399543

[ref42] Yue B. Zong G. Tao R. Wei Z. Lu Y. (2022). Crosstalk between traditional Chinese medicine-derived polysaccharides and the gut microbiota: a new perspective to understand traditional Chinese medicine. Phytother. Res. 36, 4125–4138. doi: 10.1002/ptr.7607, PMID: 36100366

[ref43] Zhan Y. Wen Y. Du L. J. Wang X. X. Tang S. Y. Kong P. F.. (2022). Effects of Maren pills on the intestinal microflora and short-chain fatty acid profile in drug-induced slow transit constipation model rats. Front. Pharmacol. 13:804723. doi: 10.3389/fphar.2022.804723, PMID: 35496291 PMC9039019

[ref44] Zhang H. Y. Tian J. X. Lian F. M. Li M. Liu W. K. Zhen Z.. (2021). Therapeutic mechanisms of traditional Chinese medicine to improve metabolic diseases via the gut microbiota. Biomed. Pharmacother. 133:110857. doi: 10.1016/j.biopha.2020.110857, PMID: 33197760

