# Supplementary material for: Guiren Runchang granules alleviate slow transit constipation in mice by modulating gut microbiota and short-chain fatty acids
Source: Front Microbiol. 2025 Sep 29;16:1615297. doi: 10.3389/fmicb.2025.1615297 (PMC12515827; doi:10.3389/fmicb.2025.1615297)
Supplement: Supplementary file 1 [file Table_1.DOCX]

Table S1. Herbal constituents of GRG.

| **Pharmaceutical name** | **Amount (g)** |
| --- | --- |
| Angelica | 10 |
| Raw atractylodes | 25 |
| Peach kernel | 15 |
| Cistanche | 15 |
| Fructus Auranti | 25 |
| Magnolia | 10 |
| Pollen, Typhae | 15 |
| Trogopterus dung | 12 |
| Trichosanthes kirilowii maxim | 20 |
| Liquorice dry powder | 6 |

Table S2. Anosim intergroup difference analysis

| Group 1 | Group 2 | R | p-value |
| --- | --- | --- | --- |
| NC | STC | 0.925 | <0.01 |
| LD | STC | 0.815 | <0.01 |
| MD | STC | 0.837 | <0.01 |
| HD | STC | 0.729 | <0.01 |
| MO | STC | 0.937 | <0.01 |
